# Handover Practices for Psychiatric Admissions: A Retrospective Review of Communication Gaps

**DOI:** 10.1192/bjo.2025.10469

**Published:** 2025-06-20

**Authors:** Aalap Asurlekar

**Affiliations:** NHS Lanarkshire, Glasgow, United Kingdom

## Abstract

**Aims:** This study assesses the frequency and adequacy of handovers for newly admitted patients in a community psychiatry hospital, focusing on formal communication to the duty doctor.

**Methods:** This study was conducted at Udston Hospital within NHS Lanarkshire, which comprises two older adult wards, each with a capacity of 20 beds. Patients, primarily aged 65 and above, were admitted either informally or under the Mental Health Act.

Data from 50 randomly selected patient admissions between January 2024 and January 2025 were collected using the electronic patient record platform MORSE. Handover was defined as any documented verbal or written communication to the duty doctor regarding a patient’s admission. Categorical data analysis was performed to identify trends in handover practices.

**Results:** The study revealed significant deficiencies in handover communication, with 54% of patients admitted without a formal handover. Home was the most common admission source (70%), with an even split in handover rates (51.4% handed over vs 48.6% not handed over). In contrast, hospital admissions had the lowest handover rate, 71.4% not handed over, suggesting direct transfers without a formal process in most cases. Care home admissions were also less likely to involve a handover with 62.5% not being handed over. Regarding detention status, 56.7% of informal patients were not handed over. In contrast, all patients under a Community Treatment Order (CTO) were handed over (100%), likely due to legal requirements for coordinated care. Patients under Short-Term Detention Certificates (STDC) and Emergency Detention Certificates (EDC) had a near-equal split in handover rates. These findings suggest that handover processes are more structured for detained patients but remain inconsistent for informal admissions and transfers from hospitals and care homes.

**Conclusion:** Inconsistent handover practices for new admissions highlight a critical gap in communication. Findings highlight the urgent need for standardized handover protocols, including mandatory documentation for all admissions, to enhance patient safety and care continuity. Implementing structured communication frameworks, such as SBAR (Situation, Background, Assessment, Recommendation), may enhance handover reliability and reduce patient safety risks.

Improving handover communication is critical to minimizing patient safety risks and ensuring seamless transitions of care, particularly in psychiatric settings where detailed histories and individual care requirements are crucial. The absence of a structured handover posed risks of fragmented care, delayed treatment initiation, and insufficient awareness of patient-specific needs.

Future research should investigate barriers to effective handovers and evaluate interventions that improve adherence and patient outcomes in psychiatric settings.

